# Albumin Levels in Tear Film Modulate the Bioavailability of Medically-Relevant Topical Drugs

**DOI:** 10.3389/fphar.2019.01560

**Published:** 2020-01-28

**Authors:** Lionel Sebbag, Leah M. Moody, Jonathan P. Mochel

**Affiliations:** ^1^Department of Veterinary Clinical Sciences, College of Veterinary Medicine, Iowa State University, Ames, IA, United States; ^2^Department of Biomedical Sciences, College of Veterinary Medicine, Iowa State University, Ames, IA, United States; ^3^Lloyd Veterinary Medical Center, College of Veterinary Medicine, Iowa State University, Ames, IA, United States

**Keywords:** albumin, blood-tear barrier, protein-binding, dog, bioavailability, ocular surface

## Abstract

The breakdown of blood-tear barrier that occurs with ocular pathology allows for large amounts of albumin to leak into the tear fluid. This process likely represents an important restriction to drug absorption in ophthalmology, as only the unbound drug is transported across the ocular tissue barriers to exert its pharmacologic effect. We aimed to investigate the effects of albumin levels in tears on the bioavailability of two commonly used ophthalmic drugs: tropicamide, an antimuscarinic that produces mydriasis and cycloplegia, and latanoprost, a PGF2α analog used for the treatment of glaucoma. Eight female beagle dogs underwent a randomized, vehicle-controlled crossover trial. For each dog, one eye received 30 µl of artificial tears (control) or canine albumin (0.4 or 1.5%) at random, immediately followed by 30 µl of 1% tropicamide (2 days, 24 h washout) or 0.005% latanoprost (2 days, 72 h washout) in both eyes. Pupil diameter (digital caliper) and intraocular pressure (IOP; rebound tonometry) were recorded at various times following drug administration (0 to 480 min) and compared between both groups with a mixed model for repeated measures. Albumin in tears had a significant impact on pupillary diameter for both tropicamide (*P ≤* 0.001) and latanoprost (*P ≤* 0.047), with no differences noted between 0.4% and 1.5% concentrations. Reduction in the maximal effect (pupil size) and overall drug exposure (area under the effect time-curve of pupil size over time) were significant for tropicamide (6.2–8.5% on average, *P ≤* 0.006) but not for latanoprost (*P* ≥ 0.663). The IOP, only measured in eyes receiving latanoprost, was not significantly impacted by the addition of either 0.4% (*P* = 0.242) or 1.5% albumin (*P* = 0.879). Albumin in tear film, previously shown to leak from the conjunctival vasculature in diseased eyes, may bind to topically administered drugs and reduces their intraocular penetration and bioavailability. Further investigations in clinical patients and other commonly used ophthalmic medications are warranted.

## Introduction

Topical instillation is the most common route of drug administration in ophthalmology, especially for the treatment of anterior segment diseases (Gaudana et al., 2010). This mode of administration is convenient and non-invasive, although drug bioavailability is generally poor (typically < 10%) due to physiological, structural, and biochemical barriers to drug penetration into the eye (Järvinen et al., 1995; Gaudana et al., 2010; Bucolo et al, 2012). Upon instillation, an eyedrop is immediately diluted in the tear film and a large portion of the drug is lost through reflex tearing, nasolacrimal drainage, and systemic absorption. Residual drug has to cross ocular tissue barriers (*i.e.* cornea, sclera, and conjunctiva) to reach targets within the globe (Prausnitz and Noonan, 1998; Gaudana et al., 2010; Bucolo et al, 2012). In general, small lipophilic drugs permeate through the cornea while larger or hydrophilic compounds permeate through the conjunctiva and sclera (Prausnitz and Noonan, 1998). Protein binding in tear film represents another important restriction to drug absorption, as only the unbound drug is transported across the tissue barriers (Mikkelson et al., 1973a). In fact, the presence of albumin in tears can dramatically reduce the bioavailability of topical drugs *via* protein-drug interactions, as previously shown for pilocarpine in rabbit eyes (Mikkelson et al., 1973a).

Albumin is a relatively large (66 kDa) and negatively charged protein that is widely distributed in the body. Given the protein’s remarkable capacity for binding ligands (de Wolf and Brett, 2000), albumin serves as a reservoir and transporter for drugs and other molecules such as hormones, metabolites, and nutrients. At the level of the eye, plasma-derived albumin leaks onto the ocular surface from conjunctival vessels and mixes with the tear film (Runstrom et al., 2013). Albumin concentration in tears is generally low in healthy state but increases substantially in diseased eyes (Runstrom et al., 2013). In fact, albumin is often considered a biomarker of ocular insult or inflammation as the breakdown of blood-tear barrier noted with ocular pathology allows for large amounts of albumin to leak into the lacrimal fluid (Anderson and Leopold, 1981; Woodward and Ledgard, 1985; Runstrom et al., 2013). A recent study by Sebbag et al. showed that canine eyes with diverse ocular diseases (*e.g.* corneal ulcer, uveitis, glaucoma) had lacrimal albumin levels that were up to 14.9-fold greater than contralateral healthy eyes (Sebbag et al., 2019a).

The impact of albumin binding on the drug’s pharmacological activity is extensively studied in blood (Zhivkova, 2015), yet little is known about the physiology and function of albumin in tears or other biological fluids. In the present study, we examined the bioavailability of topically delivered drugs in the presence of clinically relevant levels of albumin in tears (Sebbag et al., 2019a). We hypothesized that the drugs’ intraocular effect will be reduced by lacrimal albumin given the inability of protein-bound drugs to permeate through ocular tissue barriers. Two ophthalmic medications were investigated as a proof-of-concept experiment: 0.005% latanoprost and 1% tropicamide. These drugs are commonly used in human and veterinary practice, and possess different physicochemical properties (*e.g.* solution pH, drug concentration) that could influence protein-drug interactions. Latanoprost, a PGF2α analog, is used for the treatment of glaucoma and ocular hypertension in human and veterinary patients (Stjernschantz, 2001; Willis et al., 2002). Tropicamide, an antimuscarinic drug, is used to achieve short-acting mydriasis for enhanced visualization of the lens, vitreous body, and fundus, as well as cycloplegia to control accommodation during the assessment of refractive error (Manny et al., 2001). Pupil response to tropicamide was also suggested as a noninvasive neurobiological test for Alzheimer’s disease and other neurodegenerative disorders (Scinto et al., 1994; Gómez-Tortosa et al., 1996), although this diagnostic test fell out of favor given large inter- and intra-individual variations and subsequent poor test specificity (Reitner et al., 1997). Drug binding to proteins in tear fluid could partly explain the aforementioned variability in pupil size, a phenomenon our group investigated in the present study to help guide future diagnostic and therapeutic applications in ophthalmology. The present work was conducted in dogs, a species that represents an excellent large animal model for translational research in humans given similarities in ocular anatomy (Vézina, 2013) and physiologic parameters pertinent to topical route of drug administration (Sebbag et al., 2019b, Sebbag et al., 2019c), as well as spontaneous disease development such as glaucoma (Grozdanic et al., 2010), dry eye (Kaswan et al., 1989) and conjunctivitis (Sebbag et al., 2019a).

## Materials and Methods

### Animals

Eight female spayed Beagle dogs (1.5–2.0 years, 7.5–10 kg) were recruited. Prior to study enrollment, dogs were part of a teaching colony and underwent weekly physical and ophthalmic examinations, including tonometry (TonoVet, Icare Finland Oy, Espoo, Finland). At study inclusion, dogs were confirmed to be healthy based on a complete physical and ophthalmic examination, including tonometry (TonoVet), Schirmer tear test-1 (STT-1; Eye Care Product Manufacturing LLC, Tucson, AZ, USA), slit-lamp biomicroscopy (SL-17; Kowa Company, Ltd., Tokyo, Japan), and indirect ophthalmoscopy (Keeler Vantage; Keeler Instruments, Inc., Broomall, PA, USA). The study was approved by the Institutional Animal Care and Use Committee of Iowa State University (protocol #19-049), and conducted in accordance with the Association for Research in Vision and Ophthalmology guidelines for animal use.

### Experiment

Two canine albumin ophthalmic solutions (0.4% and 1.5%) were formulated by mixing canine albumin lyophilized powder (Animal Blood Resource International, Stockbridge, MI) with lubricating eye drops (Artificial tears solution, Rugby, Rockville Center, NY, USA) in a sterile manner under a laminar flow hood. Of note, albumin concentrations selected herein (0.4% and 1.5%) aimed to achieve tear film albumin levels of ~ 1 to 5 mg/ml (*i.e.*, after dilution of the instilled drop with the canine tear film, accounting for ~ 3-fold dilution; Sebbag et al., 2019b), thus representing a spectrum of albumin levels in tears of dogs with spontaneous or experimentally-induced conjunctivitis (Sebbag et al., 2019a). Artificial tears solution without albumin (vehicle only) was used as control for the experiment. Albumin and vehicle solutions were kept in the refrigerator (4°C) and used within 7 days of preparation.

For each dog, one eye was randomly selected to receive albumin solutions while the contralateral eye served as control (vehicle solution); this choice was kept constant throughout the study. Tropicamide 1% (Sandoz Inc., Princeton, New Jersey, USA) and latanoprost 0.005% (Sandoz Inc., Princeton, New Jersey, USA) were each investigated over two separate days. To allow pupil size to return to baseline and avoid a carry-over effect, the washout between experimental days was 24 h for tropicamide (Rubin and Wolfes, 1962) and 72 h for latanoprost (Pirie et al., 2011). For each drug, the eye allocated to albumin was randomly assigned to receive either 4 mg/ml or 15 mg/ml albumin solution on the first experimental day, and *vice versa* on the second day.

The experiments took place in a quiet and uniformly illuminated room (500 lux) under controlled temperature (70–72°F) and ambient humidity (25–30%). Measurements of pupil diameter (PD) were obtained with a digital Vernier caliper (± 0.01 mm, Ultratech No. 1433, General Tools & Instruments, Secaucus, NJ) held adjacent to the cornea, while measurements of intraocular pressure (IOP) were obtained with rebound tonometry (TonoVet, Icare Finland Oy, Espoo, Finland). Baseline PD and IOP were recorded in both eyes of each dog at the beginning of each study day.

Using a pipette, 30 µl of experimental solution were delivered topically: albumin in one eye, vehicle in the other. This was immediately followed (< 10 sec) by topical instillation of 30 µl of the drug (tropicamide or latanoprost) in both eyes. Then, PD (tropicamide and latanoprost) and IOP (latanoprost only) were recorded in both eyes at the following time points: 2, 4, 6, 8, 10, 15, 20, 30, 45, 60, 90, 120, 180, 240, and 480 min.

### Data Analysis

Normality of data was assessed with the Shapiro-Wilk test. Differences in pupil diameter (tropicamide, latanoprost) and IOP (latanoprost) between eyes receiving vehicle (control) or albumin (0.4 or 1.5%) were assessed with a mixed model for repeated measures (MMRM) using the R software version 3.6.0 (Lee et al., 2018). In the model, PD or IOP were the response variable, the group (control or albumin), time (0 to 480 min) and group-by-time interaction were treated as fixed effects, and the animal and animal-by-group interaction were treated as random effects, using animal as block. After the model was fit, the fixed effects were tested, and comparisons between control and albumin eyes at baseline and each time point were made. The R software was also used to calculate the area under the effect-time curve (AUETC) and the maximal effect on pupil diameter (maximal dilation for tropicamide, maximal constriction for latanoprost). Paired t-tests were conducted with SigmaPlot 14.0 (Systat Software Inc., San Jose, CA, USA) to assess the following parameters: (i) AUETC for tropicamide and latanoprost, (ii) PD_max_ for tropicamide, and (iii) PD_min_ for latanoprost. *P* values < 0.05 were considered statistically significant.

## Results

Results from the Shapiro-Wilk test confirmed that the experimental data were normally distributed. Results are therefore presented as mean ± standard deviation (SD).

### Pupil Dilation From Tropicamide

**Figure 1:**
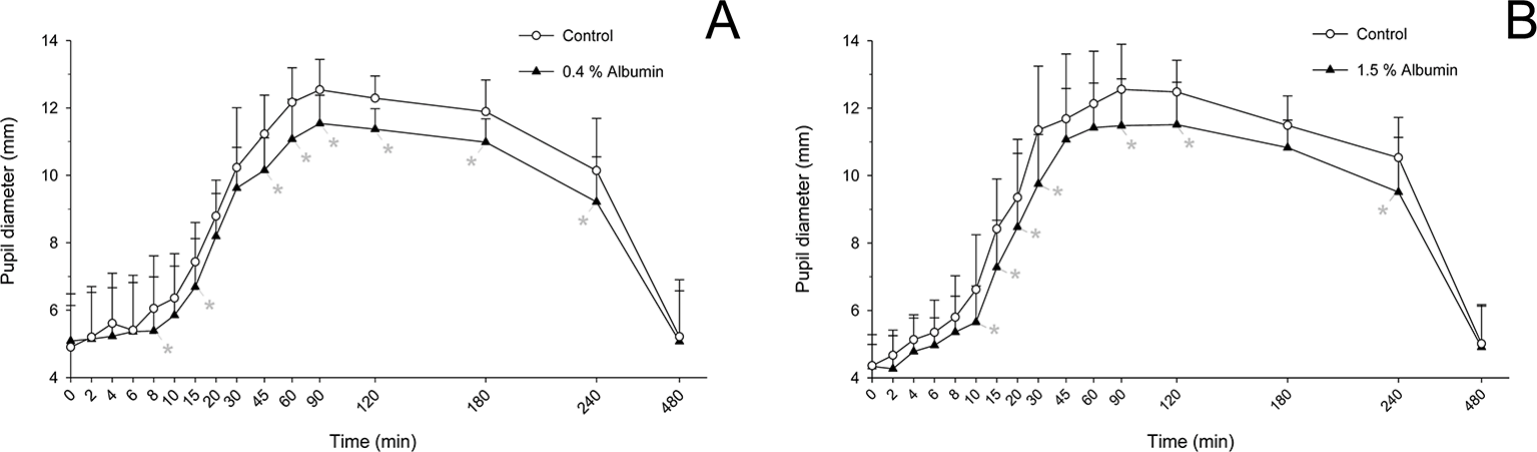
Mean + SD pupil diameter from 0 to 480 min in dogs receiving 1% tropicamide in both eyes, immediately preceded by topical instillation of artificial tears (control, white circles) in one randomly selected eye, and either 0.4% albumin (**A**, black triangles) or 1.5% albumin (**B**, black triangles) in the other eye. Statistical differences (*P* < 0.05) obtained with mixed model for repeated measures are depicted by gray asterisks (*).

**Figure 2:**
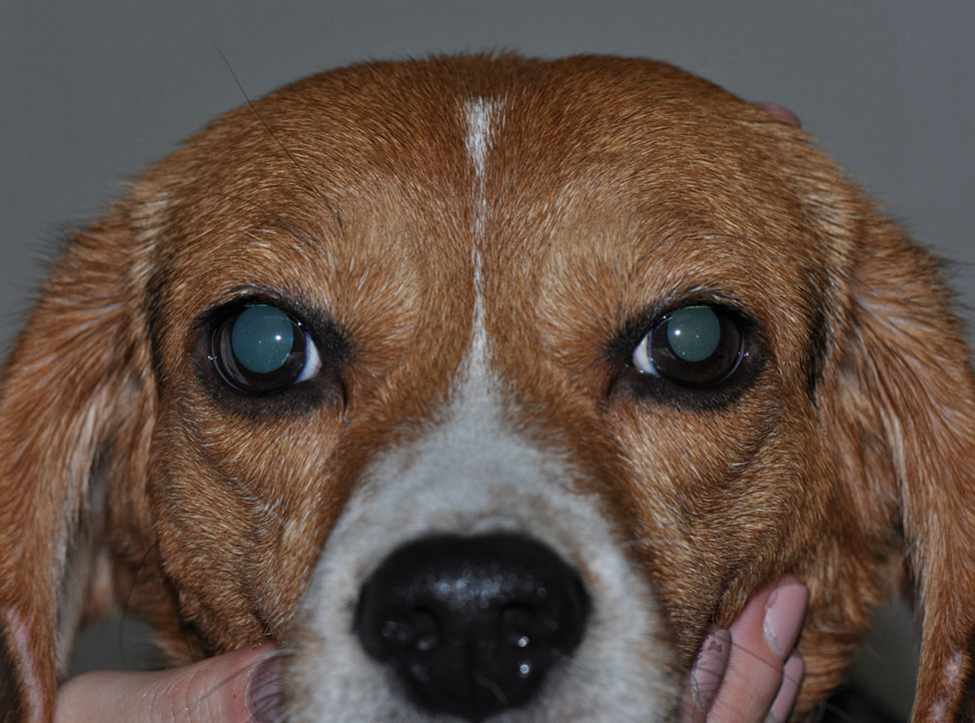
Clinical image of a Beagle dog at 45 min following topical instillation of 1% tropicamide in both eyes, immediately preceded by topical artificial tears (right eye, control) and 0.4% albumin (left eye). Note the lower degree of mydriasis in the left eye.

**Figure 3:**
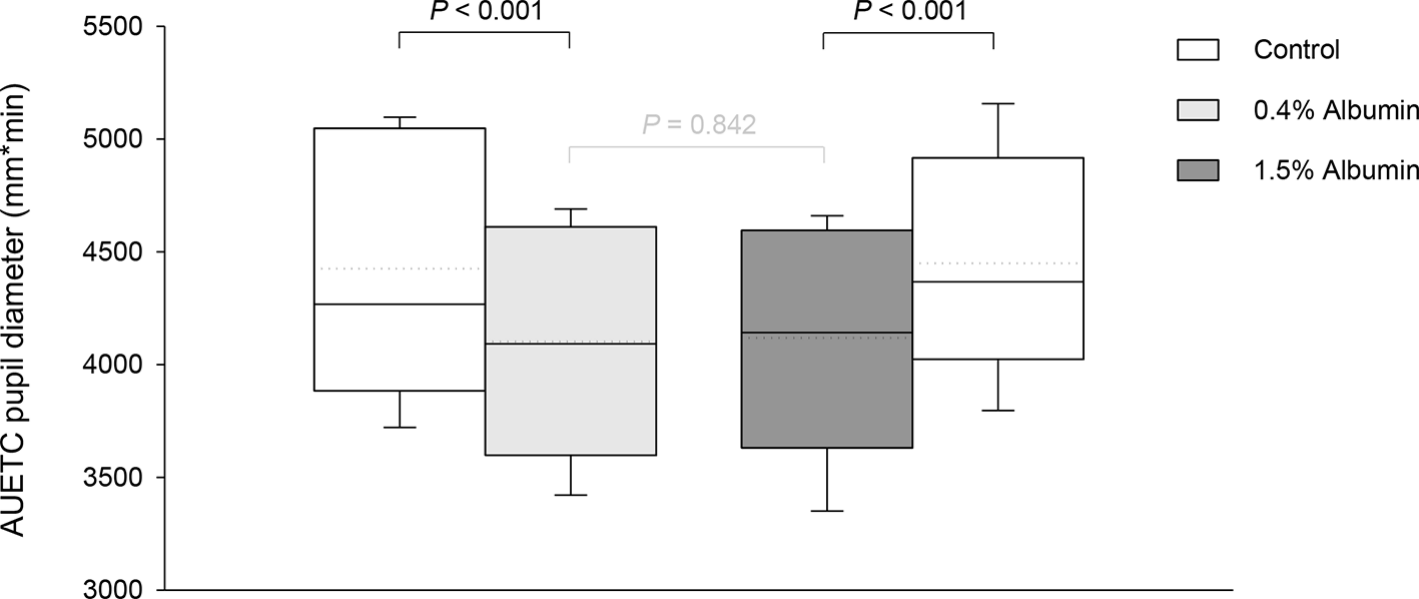
Box-and-whisker plots depicting the area under the effect-time curve (AUETC) of pupil diameter over time (0 to 480 min) in dogs receiving 1% tropicamide in both eyes, immediately preceded by topical instillation of artificial tears (control, white boxes) in one randomly selected eye, and either 0.4% albumin (light gray box) or 1.5% albumin (dark gray box) in the other eye. Mean and median values are shown by horizontal dotted and solid lines, respectively. First and third quartiles (25th and 75th percentiles) are represented by the lower and upper limits of the box, respectively, while the 2.5th and the 97.5th percentiles are shown as the lower and upper whiskers, respectively.

**Figure 4:**
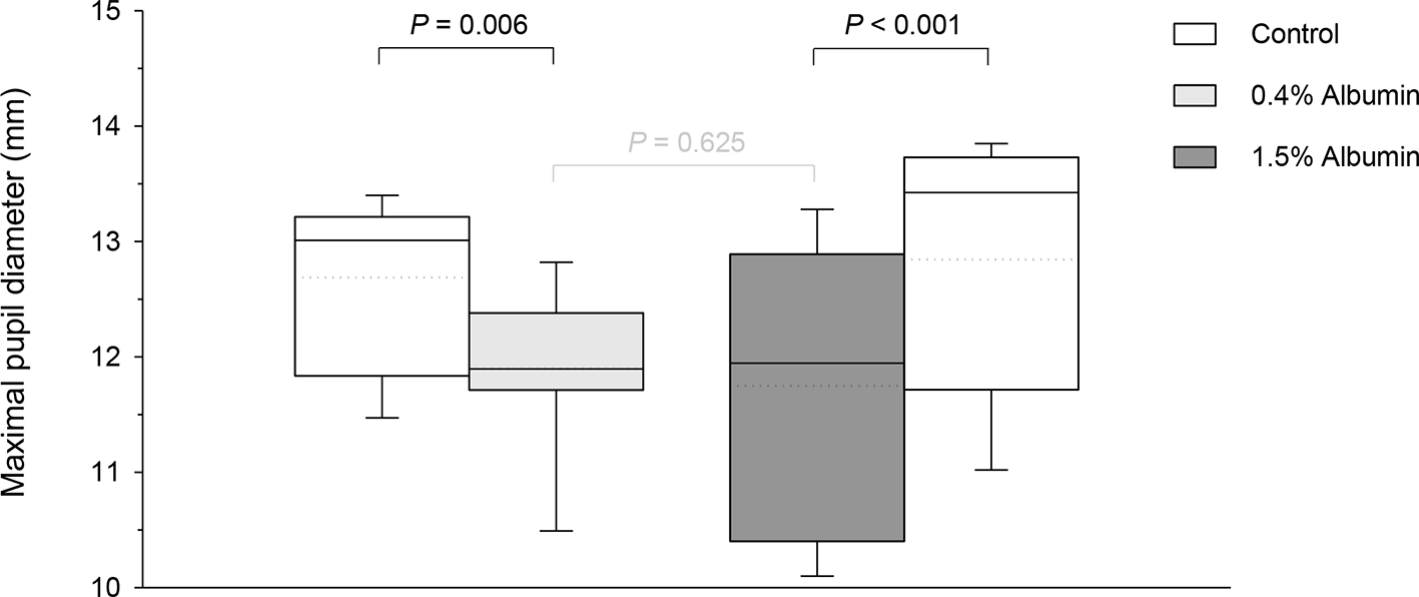
Box-and-whisker plots depicting the maximal pupillary diameter in dogs receiving 1% tropicamide in both eyes, immediately preceded by topical instillation of artificial tears (control, white boxes) in one randomly selected eye, and either 0.4% albumin (light gray box) or 1.5% albumin (dark gray box) in the other eye. Mean and median values are shown by horizontal dotted and solid lines, respectively. First and third quartiles (25th and 75th percentiles) are represented by the lower and upper limits of the box, respectively, while the 2.5th and the 97.5th percentiles are shown as the lower and upper whiskers, respectively.

Taking the variable “time” into account, albumin had a significant effect on pupil diameter post- tropicamide administration for both 0.4% (*P* = 0.001) and 1.5% concentrations (*P* = 0.001). Compared to the contralateral eye (control), pupillary dilation was significantly reduced in eyes receiving 0.4% albumin as early as 8 min (*P* = 0.043) and as late as 240 min (*P* = 0.009) (**Figure 1A**), and in eyes receiving 1.5% albumin as early as 8 min (*P* = 0.027) and as late as 240 min (*P* = 0.021) (**Figure 1B**) following instillation of 1% tropicamide. A representative clinical image is depicted in **Figure 2**, showing a lower degree of mydriasis in the dog’s left eye (0.4% albumin and 1% tropicamide) compared to the right eye (artificial tears and 1% tropicamide) at 45 min following eyedrop administration. Further, the cumulative effect of tropicamide on pupillary dilation (from 0 to 480 min) was significantly reduced with the addition of 0.4% or 1.5% albumin (*P* < 0.001), representing an average reduction in biological response of 7.1% and 7.2% compared to controls, respectively (**Figure 3**); however, no differences were noted in AUETC (pupil size over time) between both albumin concentrations (*P* = 0.842). Last, mean ± SD maximal pupillary dilation in eyes receiving tropicamide and 0.4% albumin (11.9 ± 0.7 mm) or 1.5% albumin (11.7 ± 1.3 mm) was significantly lower (*P* ≤ 0.006) compared to contralateral controls (12.7 ± 0.8 mm, and 12.8 ± 1.1 mm, respectively), representing a reduction in biological response of 6.2% and 8.5%, respectively (**Figure 4**).

### Pupil Constriction From Latanoprost

**Figure 5:**
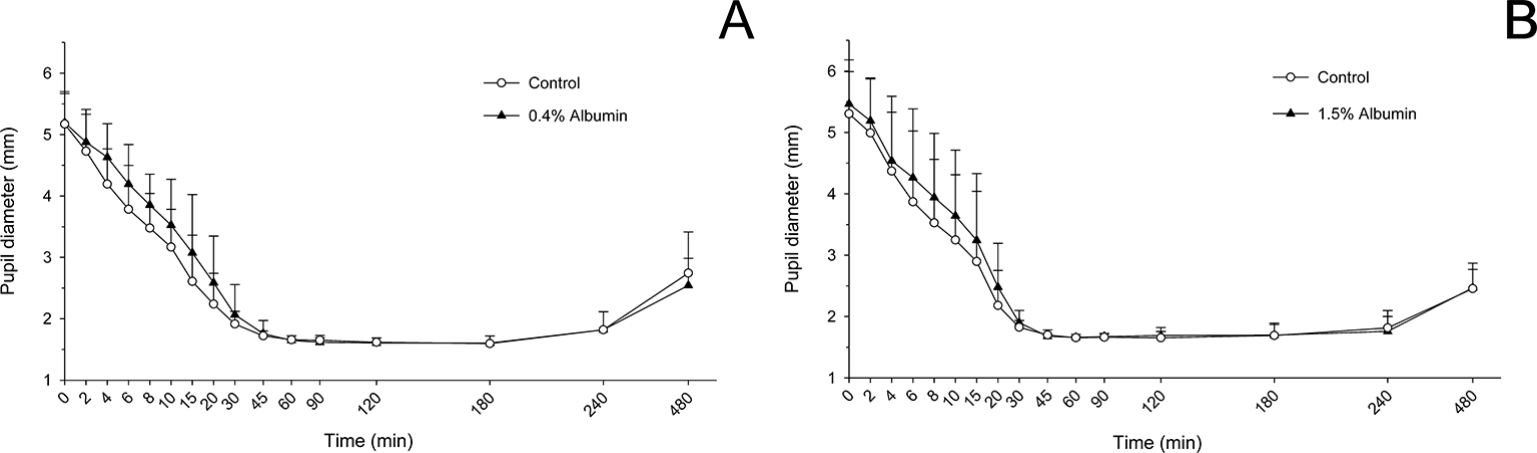
Mean + SD pupil diameter from 0 to 480 min in dogs receiving 0.005% latanoprost in both eyes, immediately preceded by topical instillation of artificial tears (control, white circles) in one randomly selected eye, and either 0.4% albumin (**A**, black triangles) or 1.5% albumin (**B**, black triangles) in the other eye. No statistical differences were noted between groups at any time point (mixed model for repeated measures, *P* ≥ 0.05).

Taking the variable “time” into account, albumin had a significant effect on pupil diameter post- latanoprost administration for both 0.4% (*P* = 0.016) and 1.5% concentrations (*P* = 0.047). Compared to the contralateral eye (control), pupillary constriction was overall reduced in eyes receiving 0.4% albumin (**Figure 5A**) or 1.5% albumin (**Figure 5B**), although differences in pupil diameter were not statistically significant at any time point following instillation of 0.005% latanoprost (*P* ≥ 0.158 and *P* ≥ 0.416, respectively). No differences were noted in AUETC (pupil size over time) between groups (*P* ≥ 0.663), nor in maximal pupillary constriction (*P* = 1.000) obtained with 0.4% albumin (1.5 ± 0.1 mm), 1.5% albumin (1.5 ± 0.2 mm), and their respective contralateral controls (1.5 ± 0.1 mm and 1.5 ± 0.2 mm).

### IOP Changes From Latanoprost

**Figure 6:**
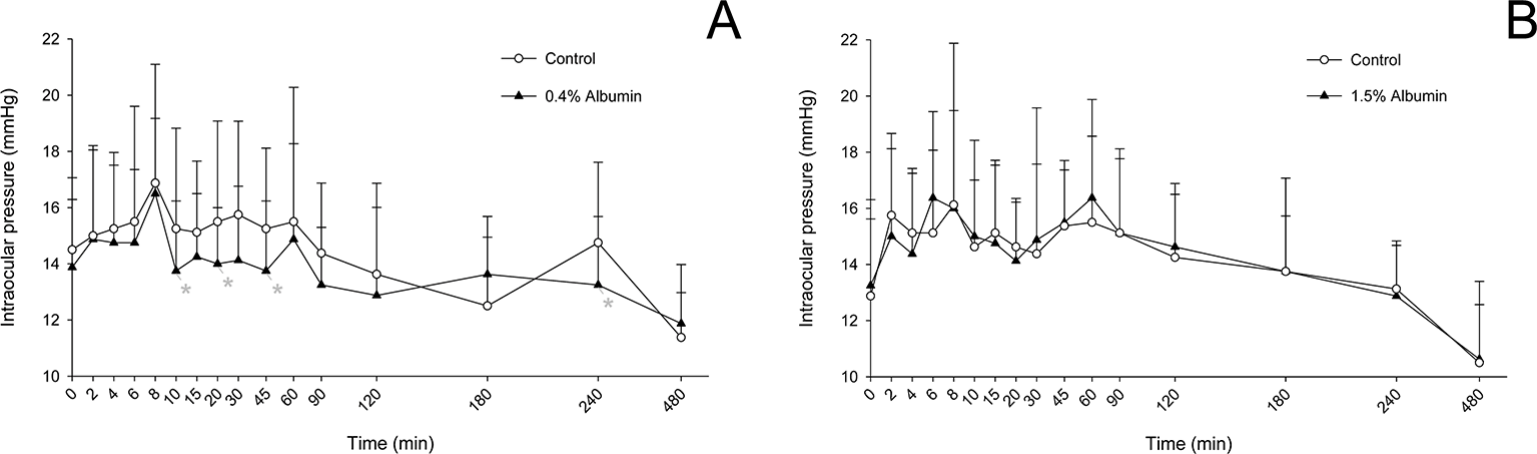
Mean + SD intraocular pressure from 0 to 480 min in dogs receiving 0.005% latanoprost in both eyes, immediately preceded by topical instillation of artificial tears (control, white circles) in one randomly selected eye, and either 0.4% albumin (**A**, black triangles) or 1.5% albumin (**B**, black triangles) in the other eye. Statistical differences (*P* < 0.05) obtained with mixed model for repeated measures are depicted by gray asterisks (*).

Compared to the contralateral eye (control), IOP was significantly lower in eyes receiving 0.4% albumin at 10 min (*P* = 0.010), 20 min (*P* = 0.010), 45 min (*P* = 0.010), and 240 min (*P* = 0.010) following instillation of 0.005% latanoprost (**Figure 6A**), while no significant changes were noted at any time point (*P* ≥ 0.262) for the 1.5% albumin group (**Figure 6B**). However, after taking “time” into account in the mixed effects model, it is important to note that the impact of albumin on IOP was not statistically significant for either 0.4% (*P* = 0.242) or 1.5% concentration (*P* = 0.879). Further, no differences were noted in AUETC (IOP over time) between control and albumin groups (*P* ≥ 0.351) or between both albumin groups (*P* = 0.979).

## Discussion

The bioavailability of ophthalmic drugs can be reduced by the presence of albumin in tears, a protein that leaks onto the ocular surface in large amounts in the diseased eye. A deeper understanding of albumin-drug interactions in tears is critical to basic researchers in pharmacology and vision science, but also physicians and veterinarians. Indeed, drug binding to albumin may partly explain the challenge of treating certain diseases in ophthalmology. For instance, a poor response of uveitis to topical corticosteroid may be due to high affinity of the drug to albumin in tears, while a poor response of infectious keratitis to topical antibiotics may be explained by the fact that only the unbound portion of an antimicrobial is microbiologically active (Dalhoff, 2018). Here, we showed a differential impact of albumin in tears on the ocular response of tropicamide and latanoprost, two common ophthalmic drugs in human and veterinary patients, and the same could be investigated in the canine model for other relevant drug classes. Dogs are particularly suited for translational research in ocular pharmacology as—unlike small laboratory animals—dogs share similar anatomical and physiological features to humans, similar environmental stressors and genetic variation, and a range of naturally occurring ophthalmic diseases that resemble the ones diagnosed in human patients.

### Ocular Response of Tropicamide and Latanoprost in the Presence of Albumin

The biological effect of tropicamide (*i.e.* mydriasis) was significantly reduced in canine eyes that received concurrent topical administration of serum albumin, regardless of the protein’s concentration. Albeit minimal (6.2–8.5%), the impact of albumin on tropicamide-induced pupillary dilation is likely underestimated compared to clinical patients given limitations inherent to the study design (described below). Interestingly, this lower degree of tropicamide-induced mydriasis was also noted in canine eyes covered with a soft contact lens, a physical barrier to drug penetration (Hatzav et al., 2016). Similar findings were noted by Mikkelson et al. in rabbit eyes receiving pilocarpine (Mikkelson et al., 1973a), although the magnitude of drug-response reduction was much greater in rabbits (75–100 fold) compared to the present study in dogs (6–8%). Such discrepancy is likely explained by two important differences in study designs. In the present experiment, the concentrations of albumin (0.4% and 1.5%) were specifically chosen based on clinically-relevant albumin levels detected in canine patients with diverse ocular diseases (Sebbag et al., 2019a), taking into account the threefold dilution effect from resident tears (Sebbag et al., 2019b) to better account for the true binding constant seen in dialysis experiments (Chrai and Robinson, 1976). In contrast, the concentrations of albumin used in rabbits were higher (1% and 3%) and may not reflect the range of biological concentrations of albumin at the ocular surface. Another key difference is the way albumin was delivered to the ocular surface. While albumin was pre-mixed with pilocarpine solution in the rabbit study, allowing for protein-drug binding to occur *ex situ* (*i.e.* away from the ocular surface) over an extended duration, albumin and tropicamide were delivered separately in the present study (albumin first, tropicamide within <10 s) so that protein-drug interactions would occur in-situ (*i.e.* in the tear film) over a short duration. From a physiological standpoint, the latter method is more appropriate as the interaction time of a topical drug to albumin in tears is generally short, limited by the rapid tear turnover rate that occurs following eyedrop administration in dogs (Sebbag et al., 2019c) or other species (Shell, 1982; Wilson, 1999). Of note, tonometry was not performed in dogs receiving tropicamide (with or without albumin) in the present study given the lack of effect of tropicamide on IOP in healthy canine eyes (Jugant et al., 2019), but this parameter could be considered in future studies as IOP can vary from tropicamide in dogs receiving sedation (Jugant et al., 2019) or dogs with glaucomatous eyes.

The pharmacological activity of latanoprost (*i.e.* miosis) was also reduced in the presence of albumin in tears, although not to the same extent as for tropicamide. Indeed, differences in pupil size between albumin and control eyes were limited in duration (up to 30 min, compared to 240 min for tropicamide) and somewhat limited in magnitude (1% non-significant change in AUETC). These findings are likely explained by the high sensitivity of the iris sphincter muscle to the drug (Yoshitomi and Ito, 1988). The minimum amount of PGF2α required to generate contraction of the iris sphincter in dogs is 10^-10^ M (Yoshitomi and Ito, 1988), while the concentration of latanoprost applied topically is approximately 10^6^ higher (0.005% ~ 10^-4^ M). The amount of drug lost to albumin in tears is therefore insignificant, as only a small fraction of intraocular drug penetration is sufficient to cause miosis. Furthermore, once latanoprost reaches the anterior chamber, the drug acts directly on the iris sphincter muscle but also indirectly through the release of endogenous prostaglandins (Yousufzai et al., 1996; Bergh et al., 2002). Endogenous PGF2α, which further acts on the prostanoid FP receptors and contributes to the sphincter muscle’s tone in dogs (Yoshitomi and Ito, 1988), is released inside the anterior chamber and is thereby not affected by albumin levels in the tear film.

Following latanoprost administration, the overall effect of lacrimal albumin on IOP values was non-significant for either albumin concentration. Compared to control eyes, a significantly lower IOP was noted at selected times (10, 20, 45, and 240 min) in eyes receiving 0.4% albumin concurrently to latanoprost, although it is important to note that IOP readings displayed a large variability within—and between—subjects in all groups. To reduce IOP variability, a recent study in healthy Beagle dogs (Fentiman et al., 2019) recommended a minimum of five training days immediately prior to the start of the study, and collecting IOP readings in triplicate at each time. In absence of such precautions, the IOP results of the present study are likely confounded by a large measurement noise.

### Factors Affecting the Impact of Albumin on Drug Bioavailability

**Figure 7:**
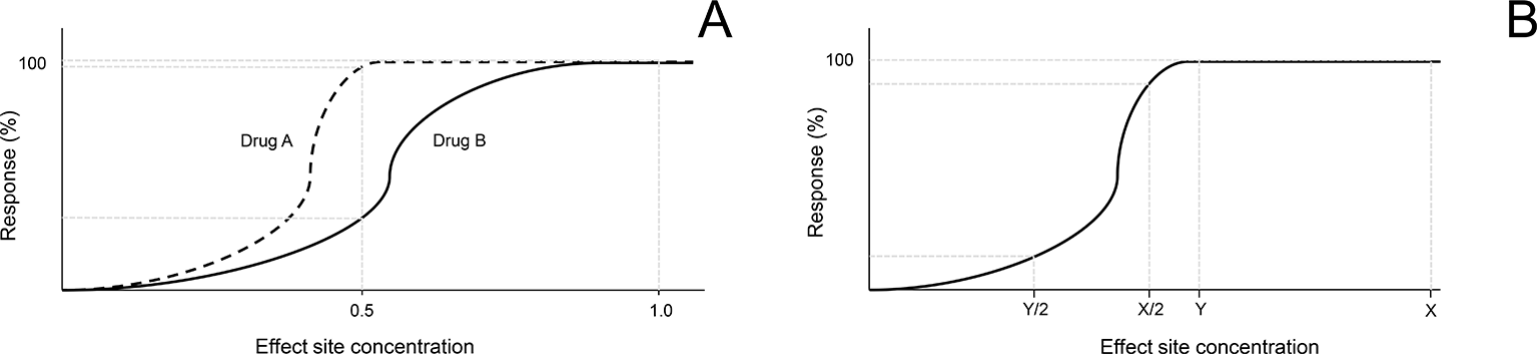
Hypothetical scenarios highlighting the importance of dose-response relationship in understanding the impact of albumin in tears on the biological activity of an ophthalmic drug. **(A)** A 50% reduction in the amount of drug that can penetrate inside the eye will have a minimal effect on the biological effect of drug A (dashed line), but a profound effect on drug B (solid line). **(B)** For the same drug, a 50% reduction in the amount of drug that can penetrate inside the eye will have a minimal effect on the biological response if the initial drug concentration was high (X), but a profound effect if the initial drug concentration was relatively low (Y).

The present findings provide evidence that albumin levels in tears does not affect all drugs in a uniform manner. Rather, the mechanism of action of a drug and/or potency for its biological target can modulate the impact of lacrimal albumin on the drug’s pharmacological activity. The dose-response relationship, a cornerstone of pharmacology/toxicology, defines the role of a dose for a chemical (*e.g.* drug, toxic agent) in evoking biological response (Snyder, 1984). **Figure 7A** depicts two drugs (A and B) with different dose-response profiles. In this scenario, if lacrimal albumin reduces the amount of free drug available inside the eye by 50%, the response observed (*e.g.* pupillary dilation) will be greatly reduced for drug B but minimally affected for drug A. Along the same line, the amount of drug applied topically is an important factor to take into consideration. For a given drug, if the dose administered topically falls in the “far right” of the drug’s dose-response curve, a reduction in drug available after albumin binding would only minimally affect the observed response (dose X, **Figure 7B**); in contrast, if the dose delivered produces an effect that falls within the steep portion of the dose-response curve, the impact of dose reduction from albumin would be more pronounced (dose Y, **Figure 7B**). Mikkelson and colleagues showed that the entire biological response (pilocarpine-induced miosis) could be suppressed when low drug concentrations were used in the presence of serum albumin (Mikkelson et al., 1973a). Future studies leveraging this work should assess the ocular response obtained from different concentrations of the same drug (*e.g.* tropicamide 0.05%, 0.5%, 1%, 2%) in the presence of albumin in tears.

A number of other factors can influence the drug-protein interactions on the ocular surface, including albumin concentrations in tears and physicochemical properties of the individual drug. In plasma, higher levels of albumin can further reduce the biological response of a drug, as exemplified by lower antimicrobial activity of fluoroquinolones with increasing levels of serum albumin (Zeitlinger et al., 2008). In tears, however, the present study did not find statistical differences between 0.4% and 1.5% albumin for either tropicamide or latanoprost. It is possible that the magnitude of albumin levels is not as critical in tears as it is in plasma, as lacrimal concentrations of albumin are relatively small (< 2%) (Sebbag et al., 2019a) in comparison to blood (≥ 4%) (Zeitlinger et al., 2008). Similarly, the influence of molecular weight on drug-albumin affinity may be minimal in tears, as most ophthalmic drugs have a relatively small and narrow range of molecular weights (e.g. 284 Da for tropicamide, 432 Da for latanoprost). In contrast, the pH of ophthalmic drugs is likely more impactful: pH varies from one ophthalmic solution to another, and albumin is known to change its binding affinity and conformation when exposed to changes in solution pH (Kochansky et al., 2008). Here, tropicamide solution is slightly more acidic (pH 5.8) than latanoprost solution (pH 6.7), although both solutions are near physiologic pH for the ocular surface and may have minimal impact on albumin affinity compared to other ophthalmic drugs such as dorzolamide (pH 4.5). Future studies should investigate the importance (or lack thereof) of other relevant biological factors on albumin-drug interactions in tear fluid, such as drug lipophilicity, viscosity, temperature at the ocular surface, fatty acids levels in tears, and drug-drug interactions (Zeitlinger et al., 2011).

### The Study May Underestimate Drug-Proteins Interactions and Their Impact on Bioavailability

The present work likely underestimates the true impact of proteins on ocular bioavailability of drugs, as the study design has two noteworthy limitations. First, although the lag time between albumin and drug instillation was short (< 10 s), it may be sufficient for a portion of the administered albumin to be washed out of the ocular surface by the time the drug mixes with the tear film, as most of an administered eyedrop is lost to drainage in the first 15 to 30 s (Shell, 1982; Wilson, 1999). Second, although albumin is a major actor of drug-protein binding on the ocular surface, other proteins play a critical role too. Using equilibrium dialysis, Chrai and Robinson showed that sulfisoxazole primarily binds to albumin in tears, but also α-globulin and (to a lesser extent) γ-globulin and lysozyme (Chrai and Robinson, 1976), all of which are normal components of tears in dogs and other species. Ultimately, the authors recommend that future investigations be conducted with experimental models of blood-tear barrier breakdown, such as histamine-induced conjunctivitis in dogs (Sebbag et al., 2019a). With such models, albumin and other key proteins are already present on the ocular surface when the drug is administered topically, although individual protein concentrations may vary from one eye to another, and this variability should be accounted for in the interpretation of study results. Furthermore, such models would account for other important changes that occur with ocular surface inflammation and that could affect drug-protein interactions, such as altered tear volume and turnover rate, tear film instability, and variations in mucin composition (Rolando and Zierhut, 2001).

### Strategies to Minimize Drug-Proteins Interactions and Enhance Ocular Bioavailability

A few strategies can be used to minimize the effects of drug-protein interactions on the ocular surface, and thereby maximize the drug pharmacological action by enhancing intraocular bioavailability. First, a higher drug concentration should be considered, especially if available commercially (*e.g.* tropicamide 1% instead of 0.5%), as the resulting concentration gradient of unbound drug will be higher (Fick’s first law of diffusion). Second, the amount of protein leakage into the tear film can be reduced by stabilizing the blood-tear barrier in the diseased eye, a process achieved by treating the underlying ocular disease (Sebbag et al., 2019a) and/or using vasoprotective drugs such as calcium dobesilate (van Bijsterveld and Janssen, 1981). Last, drug-protein interactions can be reduced by using competitive inhibitors of protein binding; for instance, Mikkelson and colleagues showed that the biological activity of pilocarpine (a miotic agent) increased 10-fold in the presence of the competitive inhibitor cetylpyridinium chloride (Mikkelson et al., 1973b). However, the use of competitive inhibition of albumin binding is discouraged until the importance of albumin on the ocular surface is fully elucidated. In fact, albumin in tear film may serve as a double-edged sword, being detrimental to the ocular bioavailability of topically administered medications, but also beneficial for symptomatic relief of dry eye (Shimmura et al., 2003; Seki et al., 2015), corneal wound healing (Shimmura et al., 2003), and anti-oxidative and anti-inflammatory activities (Merlot et al., 2014).

### Conclusion

Albumin in tears modulate the ocular bioavailability of topically administered drugs, as observed in a “large animal” model that shares similar anatomical and physiological features with humans. The effect of albumin depends on the medication (*e.g.* drug concentration and inherent physicochemical properties) and was overall mild (< 10%) in the present work on healthy canine eyes, albeit likely underestimated given the rapid tear turnover rate following eyedrop administration. Models of ocular surface inflammation could enable future pharmacological studies to gain a deeper understanding of protein-drug interactions, accounting for albumin leakage in tears as well as other relevant factors that affect ocular surface homeostasis.

## Data Availability Statement

The datasets generated for this study are available on request to the corresponding authors.

## Ethics Statement

The animal study was reviewed and approved by the Institutional Animal Care and Use Committee of Iowa State University.

## Author Contributions

LS conceptualized and designed the study in consultation with JM. LS and LM performed the experiments. LS and JM analyzed the data. All authors wrote the manuscript.

## Conflict of Interest

The authors declare that the research was conducted in the absence of any commercial or financial relationships that could be construed as a potential conflict of interest.
